# An Algorithm for Strapdown Airborne Gravity Disturbance Vector Measurement Based on High-Precision Navigation and EGM2008

**DOI:** 10.3390/s24185899

**Published:** 2024-09-11

**Authors:** Ke Fang, Tijing Cai

**Affiliations:** School of Instrument Science and Engineering, Southeast University, Nanjing 210096, China; fangke@seu.edu.cn

**Keywords:** EGM2008 model, error separation and compensation, gravity disturbance vector measurement, strapdown navigation algorithm

## Abstract

Attitude errors, accelerometer bias, the gravity disturbance vector, and their coupling are the primary factors obstructing strapdown airborne vector gravimetry. This paper takes the geocentric inertial frame as a reference and solves the kinematic equations of its motion and its errors of the body frame and local geographic frame in the Lie group, respectively; the attitude accuracy is improved through a high-precision navigation algorithm. The constant accelerometer bias is estimated through Kalman filtering and is deducted from the accelerometer output to eliminate its influence. Based on the EGM2008 model, the low-frequency components of the gravity disturbance vector are corrected. The gravity disturbance vectors after model data fusion were low-pass filtered to obtain the ultimate results. This method was applied to flight experimental data in the South China Sea, and a gravity anomaly accuracy of better than 0.5 mGal, a northward gravity disturbance accuracy of 0.85 mGal, and an eastward gravity disturbance accuracy of 4.0 mGal were obtained, with a spatial resolution of approximately 4.8 km.

## 1. Introduction

The Earth’s gravitational field is a crucial inherent physical property, and precise determination of the gravity disturbance vector is of great significance in geodesy, space science, geodynamics, resource exploration, and high-precision navigation and guidance. Among various gravity measurement techniques, strapdown airborne gravimetry stands out as an accurate and efficient method for obtaining high-resolution gravity disturbance vectors [1,2,3,4].

In the 1990s, Gleason [5] and Jekeli [6] explored the potential of using a Strapdown Inertial Navigation System (SINS) and Global Navigation Satellite System (GNSS) for gravity disturbance vector measurement. It was demonstrated that with relatively stable uncompensated gyroscope drift and reasonable modeling of gravity disturbance vectors, certain frequency bands of the horizontal gravity disturbance vector could be measured. Jekeli and Kwon [7,8] introduced a novel method for airborne gravity disturbance vector measurement based on SINS/Global Positioning System (GPS)-integrated systems. Their method used motion acceleration as the measurement and constructs a Kalman filter in the inertial coordinate system to calculate the gravity disturbance vector from the residuals of estimated inertial sensor errors and misalignment angle errors. Data from the University of Calgary resulted in a horizontal component accuracy of approximately 6 mGal and a vertical component accuracy of 3–4 mGal (note: mGal is a unit of acceleration commonly used in geophysics and gravimetry, $1mGal=10^{-5}m/s^{2}$). Attitude errors, accelerometer bias, the gravity disturbance vector, and their couplings are the main factors affecting the measurement accuracy in strapdown airborne gravity disturbance vector measurement. To eliminate low-frequency system errors in strapdown gravity disturbance measurement results, Cai et al. [9] proposed a method for determining the horizontal components of the gravity disturbance vector based on the Earth Gravitational Model 2008 (EGM2008). Additionally, some scholars suggest that reasonable modeling of the gravity disturbance vector is an effective means of distinguishing between accelerometer bias and the gravity disturbance vector [10,11]. Commonly used statistical models for gravity disturbance include the exponential anomaly model [12], Bessel anomaly model [13], second-order Markov anomaly model [14], and third-order Markov undulation model [15]. For processing a set of parallel survey lines, Bolotin and Vyazmin [16] proposed a new model to describe a two-dimensional local gravity field, considering the spatial correlation between the survey lines. In 2022, Cai et al. [17] proposed a two-step method which separates attitude error and accelerometer bias in the navigation algorithm. The compensated specific force in the local geographic coordinate system (*n*-frame) is substituted into the strapdown gravity disturbance vector calculation equation in which the gravity disturbance vector is modeled as a second-order Markov stochastic process to construct a gravity disturbance vector solution filtering model, and the gravity disturbance vector is estimated through Kalman filtering and smoothing. Despite these advancements, challenges remain, such as determining whether the local gravity disturbance vector conforms to the characteristics of an isotropic plane random field and understanding how parameters like the correlation distance of the gravity disturbance vector and the intensity of white noise driving the Markov stochastic processes impact the model. Furthermore, improving attitude calculation accuracy and separating accelerometer bias from the gravity disturbance vector itself are topics that warrant further research.

Among the various factors that affect the accuracy of strapdown gravity disturbance vector measurement, the coupling error between attitude error and vertical gravity accel-eration is one of the key obstacles to accurate horizontal gravity disturbance measurement [6]. High-precision attitude determination is also a critical issue in the SINS algorithm. In recent years, methods based on Lie groups and Lie algebras have gained significant attention in SINS attitude determination. These methods leverage the mathematical properties of Lie groups, such as the $SE\left(3\right)$ group, and Lie algebras to provide a more rigorous and effective framework for dealing with complex rotational motions and error propagation. The $SE\left(3\right)$ group offers a unified description of translational and rotational motions in three-dimensional space, while the Lie algebra characterizes the local properties of the Lie group, with exponential and logarithmic mappings linking the algebra to the group, enabling linear computations in the Lie algebra to reflect the nonlinear operations in the Lie group [18]. In 2016, Mao proposed a SINS navigation update algorithm based on the $SE\left(3\right)$ group [19]. For SINS error propagation, Barrau and Bonnabel introduced the invariant extended Kalman filter (IEKF) [20]. Fang et al., using the Earth-centered inertial frame as a reference, unified the SINS update algorithm and error propagation within the $SE\left(3\right)$ group, deriving an algorithm that decouples the motion of the body frame (*b*-frame) and the *n*-frame, with the propagation of motion errors in the *b*-frame being entirely independent of the global navigation parameters. This approach, relying on the mapping between the Lie group and its Lie algebra, effectively mitigates non-commutative rotation errors and coordinates frame inconsistency errors, thereby improving attitude accuracy [21].

In the context of correcting low-frequency trend errors induced by the cumulative errors of the SINS computation in gravity disturbance vector measurements, some studies [8,9,22] have employed the wavenumber correlation filtering (WCF) method to process repeated survey lines in different directions. These methods assume that highly correlated components represent gravity disturbances, while less-correlated components are considered to be measurement errors. However, in practical applications, this approach faces challenges in selecting an appropriate threshold and may inadvertently filter out some details of gravity disturbances, thereby reducing spatial resolution. Other studies [23] have utilized an endpoint-matching method to adjust the start and end points of the measurement segments, ensuring that their gravity disturbance values are aligned. This approach smooths the data of the measurement segments through linear interpolation, polynomial function interpolation, or least squares fitting, so that the gravity disturbance between the start and end points of the survey line is continuous and free from significant bias. Nevertheless, this method is highly dependent on the choice of interpolation method, and low-frequency trend errors do not necessarily follow a strict linear or polynomial function pattern. However, global gravity field models may not capture local details of the Earth’s gravity field; their trends over larger spatial scales are accurate and reliable, making them a viable option for gravity reference corrections of strapdown gravity disturbance vector measurements.

This article proposes a method for strapdown airborne gravity disturbance vector measurements based on a high-precision navigation algorithm and the EGM2008 model. Using the geocentric inertial coordinate system (*i*-frame) as a reference, the kinematic equations for the motion and motion errors of the body (*b*-frame) and the local geographic coordinate systems were separately solved in the group, thereby eliminating coordinate system inconsistency errors in the navigation update and error propagation processes. Accelerometer bias was modeled as either constant or first-order gradual to separate its influence. The low-frequency gravity disturbance vector calculated using the EGM2008 model was used to correct low frequencies in the direct-difference method’s results. Following Finite Impulse Response (FIR) filtering, the gravity disturbance vector is obtained.

## 2. Method

The basic principle of strapdown airborne gravity disturbance vector is the specific force equation of SINS [24], which is the following:

(1) $${\dot{v}}_{en}^{n}=C_{b}^{n}f^{b}-\left(\omega_{ie}^{n}+\omega_{in}^{n}\right)\times v_{en}^{n}+\gamma^{n}+\delta g^{n}$$

where $v_{en}^{n}$ is the projection of the velocity relative to the Earth in the *n*-frame, $C_{b}^{n}$ is the attitude matrix, $f^{b}$ is the specific force, $\omega_{ie}^{n}$ and $\omega_{in}^{n}$ are the projections of the angular velocities of the *e*-frame and *n*-frame relative to the *i*-frame in the *n*-frame, $\gamma^{n}$ is the normal gravity vector, and $\delta g^{n}$ is the gravity disturbance vector.

By differentiating (1), the error equation for strapdown airborne gravity disturbance vector measurement can be obtained as follows:

(2) $$d\delta g^{n}=d{\dot{v}}_{en}^{n}-C_{b}^{n}f^{b}\times \varphi -C_{b}^{n}df^{b}+\left(\omega_{ie}^{n}+\omega_{in}^{n}\right)\times dv_{en}^{n}+\left(d\omega_{ie}^{n}+d\omega_{in}^{n}\right)\times dv_{en}^{n}+d\gamma^{n}$$

where the errors of ${\dot{v}}_{en}^{n}$, $\omega_{ie}^{n}$, $\omega_{in}^{n}$, $v_{en}^{n}$, and $\gamma^{n}$ are denoted by $d{\dot{v}}_{en}^{n}$, $d\omega_{ie}^{n}$, $d\omega_{in}^{n}$, $dv_{en}^{n}$, and $d\gamma^{n}$, respectively. Apart from these error sources, attitude error φ and accelerometer bias $df^{b}$, as well as their coupling with the gravity disturbance vector itself in the Kalman filter estimation process, are the main obstacles. The following measures are proposed to improve the measurement accuracy of the gravity disturbance vector, especially the two horizontal components, for these main factors.

### 2.1. High-Precision Navigation Algorithm

Before the aircraft enters the surveying area for gravity measurements, it typically performs dynamic maneuvers to enhance the observability of navigation parameters and inertial device error parameters. However, these dynamic maneuvers can introduce significant attitude estimation errors in the SINS, primarily due to rotational non-commutativity errors caused by the aircraft’s dynamic angular motion and coordinate system inconsistency errors arising from inconsistencies between navigation parameter reference frames. In strapdown gravity disturbance vector measurements, accurate attitude estimation is crucial because a 1″ attitude error can result in approximately 4.9 mGal of horizontal gravity disturbance coupling error. This implies that attitude errors are one of the primary factors limiting the accuracy of strapdown gravity disturbance vector measurements. Moreover, gravity measurement is a common post-processing task that does not have strict requirements for real-time data processing or algorithm complexity. Therefore, this paper proposes a high-order polynomial fitting method that first integrates multi-sampling SINS outputs to fit the angular and linear motion of the aircraft. Subsequently, the SINS update algorithm and the SINS/GNSS integrated Kalman filtering are applied in the $SE\left(3\right)$ group to obtain accurate attitude. Through the SINS navigation algorithm on the $SE\left(3\right)$ group, the rotational non-commutativity errors and coordinate system inconsistency errors are compensated to maximize attitude accuracy.

The angular velocity $\omega_{ib}^{b}$ and specific force $f^{b}$ of the aircraft can be expressed as an M−1 order polynomial. By synthesizing *N* sampling data before and after the current time, according to the Principle of Linear Least Squares Estimation [25], the polynomial coefficients can be obtained using the following equation:

(3) $$a={\left(A^{T}A\right)}^{-1}A^{T}\theta$$

where $A=\left[\begin{array}{ccc} \frac{t_{2}^{M}-t_{1}^{M}}{M} & \cdots & t_{2}-t_{1}\\ \vdots & \ddots & \vdots \\ \frac{t_{N+1}^{M}-t_{N}^{M}}{M} & \cdots & t_{N+1}-t_{N} \end{array}\right]$, $t_{1},t_{2},\cdots ,t_{N}$ are the N sampling time, $\theta ={\left[\begin{array}{cccc} \theta_{1} & \theta_{2} & \cdots & \theta_{N} \end{array}\right]}^{T}$ are the N sampling data, and a is the polynomial fitting coefficient vector.

Using the quasi-stationary *i*-frame as the reference, the kinematic equations for the motions and their errors of the *b*-frame and *n*-frame are solved, respectively. The attitude $C_{b}^{i}$ of the *b*-frame relative to the *i*-frame and the velocity increment $v_{f}^{i}$ generated by the specific force acceleration $f^{b}$ are integrated into one $SE\left(3\right)$ group element, and the attitude $C_{n}^{i}$ of the *n*-frame relative to the *i*-frame and the velocity increment $v_{g}^{i}$ caused by the gravitational acceleration are formed into another $SE\left(3\right)$ group element; they are as follows [19,21,26]:

(4) $$T_{b}^{i}=\left[\begin{array}{cc} C_{b}^{i} & v_{f}^{i}\\ 0_{1\times 3} & 1 \end{array}\right]\in SE\left(3\right),\;T_{n}^{i}=\left[\begin{array}{cc} C_{n}^{i} & v_{g}^{i}\\ 0_{1\times 3} & 1 \end{array}\right]\in SE\left(3\right)$$

where the kinematic equation of $T_{b}^{i}$ is driven by the polynomial fitted $\omega_{ib}^{b}$ and $f^{b}$, while the kinematic equation of $T_{n}^{i}$ depends on the global calculated navigation parameters, namely position p and velocity $v_{en}^{n}$.

Both of the kinematic equations have the following form [26]:

(5) $$\dot{T}=T\Xi ,\Xi =\left[\begin{array}{cc} \omega \times & \rho \\ 0_{1\times 3} & 0 \end{array}\right]$$

where T is an $SE\left(3\right)$ group element that represents a motion state and Ξ is its corresponding Lie algebra that consists of angular velocity ω and linear acceleration ρ.

Then, the global navigation parameters can be updated as follows:

(6) $$\left\{\begin{array}{l} C_{b}^{n}(k)={\left[C_{n}^{i}(k)\right]}^{T}C_{b}^{i}(k)\\ v_{en}^{n}\left(k\right)=v_{en}^{n}\left(k-1\right)+{\left[C_{n}^{i}(k)\right]}^{T}\left[v_{f}^{i}\left(k\right)+v_{g}^{i}(k)\right]+a_{corr}^{n}(k)∆t\\ p\left(k\right)=p\left(k-1\right)+\frac{1}{2}\left[v_{en}^{n}\left(k-1\right)+v_{en}^{n}\left(k\right)\right]∆t \end{array}\right.$$

where k represents the time point, ∆t is the sampling interval of the SINS, $a_{corr}^{n}$ is the Coriolis term, and p is the position vector.

In the $SE\left(3\right)$ group, the errors in the aircraft’s angular and linear motions are not independent parameters in separate spaces, but are jointly represented in the Lie group space, defined as follows:

(7) $$\begin{array}{c} \delta_{b}^{i}=T_{i}^{b}{\left({\hat{T}}_{b}^{i}\right)}^{-1}=\left[\begin{array}{cc} C_{i}^{b} & v_{f}^{b}\\ 0_{1\times 3} & 1 \end{array}\right]\left[\begin{array}{cc} C_{b^{\prime }}^{i} & -C_{b^{\prime }}^{i}{\hat{v}}_{f}^{b}\\ 0_{1\times 3} & 1 \end{array}\right]\\ =\left[\begin{array}{cc} C_{b^{\prime }}^{b} & v_{f}^{b}-C_{b^{\prime }}^{b}{\hat{v}}_{f}^{b}\\ 0_{1\times 3} & 1 \end{array}\right]≜\left[\begin{array}{cc} C_{b^{\prime }}^{b} & \delta v_{f}\\ 0_{1\times 3} & 1 \end{array}\right]\in SE\left(3\right),\\ \begin{array}{c} \delta_{n}^{i}=T_{i}^{n}{\left({\hat{T}}_{n}^{i}\right)}^{-1}=\left[\begin{array}{cc} C_{i}^{n} & v_{g}^{n}\\ 0_{1\times 3} & 1 \end{array}\right]\left[\begin{array}{cc} C_{n^{\prime }}^{i} & -C_{n^{\prime }}^{i}{\hat{v}}_{g}^{n}\\ 0_{1\times 3} & 1 \end{array}\right]\\ =\left[\begin{array}{cc} C_{n^{\prime }}^{n} & v_{g}^{n}-C_{n^{\prime }}^{n}{\hat{v}}_{g}^{n}\\ 0_{1\times 3} & 1 \end{array}\right]≜\left[\begin{array}{cc} C_{n^{\prime }}^{n} & \delta v_{g}\\ 0_{1\times 3} & 1 \end{array}\right]\in SE\left(3\right) \end{array} \end{array}\;$$

where $C_{b^{\prime }}^{b}$ and $C_{n^{\prime }}^{n}$ are the Lie group invariant attitude error matrices of the *b*-frame and *n*-frame, respectively, while $\delta v_{f}$ and $\delta v_{g}$ are the Lie group invariant velocity increment errors for $v_{f}^{i}$ and $v_{g}^{i}$, respectively (note that these are errors of the velocity increments, not errors of the global velocity).

By differentiating Equation (7), the Lie group invariant error equations for the SINS can be obtained as follows:

(8) $$\begin{array}{c} {\dot{C}}_{b^{\prime }}^{b}=C_{b^{\prime }}^{b}\left(\omega_{bb^{\prime }}^{b^{\prime }}\times \right)\\ \omega_{bb^{\prime }}^{b^{\prime }}=\left(I-C_{b}^{b^{\prime }}\right){\hat{\omega }}_{ib}^{b}+C_{b}^{b^{\prime }}\delta \omega_{ib}^{b}\\ \delta {\dot{v}}_{f}=\left(I-C_{b^{\prime }}^{b}\right){\hat{f}}^{b}-C_{b^{\prime }}^{b}\left(\omega_{bb^{\prime }}^{b^{\prime }}\times \right){\hat{v}}_{f}^{b}-\delta f^{b}\\ {\dot{C}}_{n^{\prime }}^{n}=C_{n^{\prime }}^{n}\left(\omega_{nn^{\prime }}^{n^{\prime }}\times \right)\\ \omega_{nn^{\prime }}^{n^{\prime }}=\left(I-C_{n}^{n^{\prime }}\right){\hat{\omega }}_{in}^{n}+C_{n}^{n^{\prime }}\delta \omega_{in}^{n}\\ \delta {\dot{v}}_{g}=\left(I-C_{n^{\prime }}^{n}\right){\hat{g}}^{n}-C_{n^{\prime }}^{n}\left(\omega_{nn^{\prime }}^{n^{\prime }}\times \right){\hat{v}}_{g}^{n}-\delta g^{n} \end{array}$$

where ${\hat{\omega }}_{ib}^{b}$ and ${\hat{f}}^{b}$ represent the angular velocity and specific force of the *b*-frame substituted into the SINS update after polynomial fitting, with $\delta \omega_{ib}^{b}$ and $\delta f^{b}$ denoting their respective errors. Likewise, ${\hat{\omega }}_{in}^{n}$ and ${\hat{g}}^{n}$ are the angular velocity and gravitational acceleration of the *n*-frame calculated using velocity and position, with $\delta \omega_{in}^{n}$ and $\delta g^{n}$ representing their respective errors.

Similarly, Equation (8) can be expressed in the form of a Lie group differential equation shown in Equation (9), analogous to Equation (5):

(9) $$\begin{array}{c} {\dot{\delta }}_{b}^{i}=\delta_{b}^{i}\Xi_{ib}^{\delta },\Xi_{ib}^{\delta }=\left[\begin{array}{cc} \left(\omega_{bb^{\prime }}^{b^{\prime }}\times \right) & \delta a_{f}\\ 0_{1\times 3} & 0 \end{array}\right],\delta a_{f}=C_{b}^{b^{\prime }}\delta {\dot{v}}_{f},\\ {\dot{\delta }}_{n}^{i}=\delta_{n}^{i}\Xi_{in}^{\delta },\Xi_{in}^{\delta }=\left[\begin{array}{cc} \left(\omega_{nn^{\prime }}^{n^{\prime }}\times )\right. & \delta a_{g}\\ 0_{1\times 3} & 0 \end{array}\right],\delta a_{g}=C_{n}^{n^{\prime }}\delta {\dot{v}}_{g} \end{array}$$

Thus, the integration filter for SINS and GNSS has been established, and the system model is as follows:

(10) $$\left\{\begin{array}{l} x=\left(\delta_{b}^{i},\delta_{n}^{i},\delta p,\varepsilon ,\nabla \right)\in SE\left(3\right)\times SE\left(3\right)\times R^{9}\\ \dot{x}=f\left(x\right)+g(w) \end{array}\right.$$

where x represents the state variable, which includes the $SE\left(3\right)$ group-invariant errors $\delta_{b}^{i}$ and $\delta_{n}^{i}$, the position error vector δp, the gyro drift vector ε, and the accelerometer bias vector ∇. f is the state transition function of x, with the transition functions for $\delta_{b}^{i}$ and $\delta_{n}^{i}$ determined by Equations (8) and (9), respectively. The state transition function for δp is governed by the invariant velocity errors, while ε and ∇ are typically modeled as constant values. g is the noise input function determined using Equation (8), and w denotes the noise vector, which includes the random noise from the gyroscopes and accelerometers.

In addition, the measurement model is as follows:

(11) $$z=\left[\begin{array}{c} v_{en,SINS}^{n}-v_{en,GNSS}^{n}\\ p_{SINS}-p_{GNSS} \end{array}\right]$$

where z is the measurement vector including velocity errors and position errors, and the subscripts ‘SINS’ and ‘GNSS’ represent the navigation computed values from the SINS and the measurements provided by the GNSS, respectively.

Based on the filtering model in Equations (10) and (11), the $SE\left(3\right)$ group-invariant errors of the SINS can be estimated using Kalman filtering and its nonlinear variants, which allows for the precise determination of the attitude and specific force in the *n*-frame.

**Figure 1:**
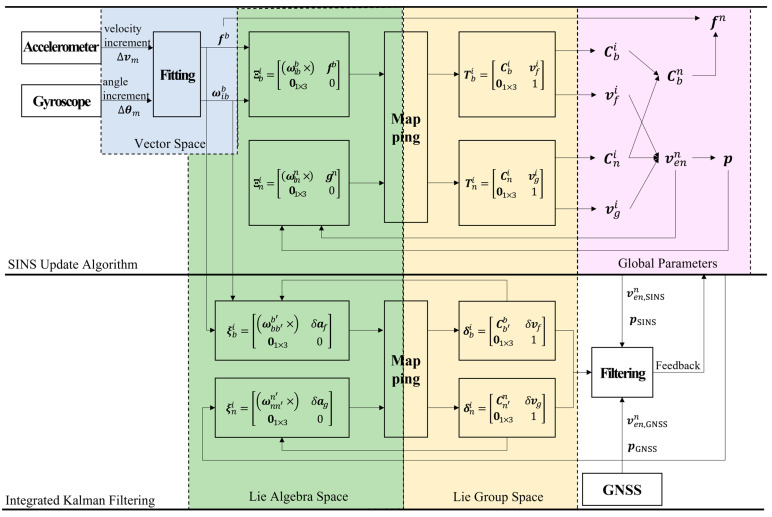
A high-precision navigation algorithm diagram. (Note: (1) The specific force and angular velocity of the aircraft is obtained through fitting the outputs of inertial sensors. (2) The motion of both the *b*-frame and the *n*-frame, along with their motion errors, are represented in the Lie group space. (3) The motion updates of the *b*-frame and *n*-frame, as well as the propagation of their motion errors, are accomplished through the mapping between Lie algebra space and Lie group space (as shown in Equation (5)). (4) The Lie group invariant errors of the *b*-frame and *n*-frame are estimated using the system model (as shown in Equations (8)–(11)) and the Kalman filter, and are subsequently fed back into the global navigation parameters).

In summary, the schematic diagram of the proposed high-precision navigation algorithm is shown in Figure 1. Referring to the diagram with the algorithm derivation, the following advantages of the algorithm can be concluded:

1. Using the least squares principle, the motion of the aircraft is approximated with polynomial fitting to closely match the actual maneuvers, thereby reducing the impact of the inertial sensor’s random noise.
2. In traditional SINS algorithms, attitude and its errors are represented in the $SO\left(3\right)$ differential manifold space, while velocity and its errors are located in three-dimensional Euclidean space, which means that the aircraft’s linear and angular motions are represented in different spaces. The joint representation based on the $SE\left(3\right)$ group ensures that both aircraft motion and its errors are represented in the same space, and both the Lie group elements representing the motion and those representing the motion errors can be updated or propagated through the exponential and logarithmic mappings between the Lie group and its associated Lie algebra. Specifically, the $SE\left(3\right)$ group-based joint representation of attitude and velocity overcomes coordinate system inconsistency errors in the SINS update process, while the joint representation of attitude and velocity errors mitigates coordinate system inconsistency errors in the error propagation process [20,21].
3. Attributed to the joint representation of aircraft angular and linear motion provided by the $SE\left(3\right)$ group, the mapping between Lie group elements and their associated Lie algebras effectively compensates for high-order coning and sculling errors in SINS update calculations, without the need to reduce the update frequency as in multi-sample algorithms.
4. By selecting a quasi-stationary *i*-frame as the reference, the motions and errors of the *b*-frame and *n*-frame are updated and propagated separately to achieve the separation and decoupling of inertial sensor errors and global navigation parameter errors. This means that the kinematic equations of the *b*-frame motion and its errors are determined using the SINS outputs and their errors, while the kinematic equations of the *n*-frame motion and its errors are completely determined using the global position and velocity. This decoupling grants global independent propagation characteristics to the *b*-frame motion errors, meaning that the kinematics of the *b*-frame errors are completely independent of the global parameters [21].

Based on the aforementioned improvements, the navigation algorithm in the $SE\left(3\right)$ group achieves higher precision in attitude estimation, which further enhances the accurate determination of specific forces in the *n*-frame and mitigates the coupling errors of horizontal gravity disturbances induced by attitude errors.

### 2.2. Separation and Compensation of Accelerometer Zero Bias

Before entering the airborne gravity disturbance vector measurement phase, the aircraft typically experiences various motion states such as acceleration, climbing, circling, hovering, and steady flight, which provide the integrated navigation filter with abundant motion information. Accelerometers used for gravity measurement tasks exhibit high precision, low noise levels, and exceptional zero-bias stability. After thorough calibration and system error compensation, the accelerometer error model can be described using the following equation:

(12) $$\nabla =\nabla_{r}+n_{a}$$

where $\nabla_{r}$ represents the random zero-bias, which varies randomly each time it is used and is estimated in the integrated navigation filter. $n_{a}$ denotes the accelerometer’s random white noise. On the one hand, polynomial fitting of the accelerometer data can partially mitigate this noise. On the other hand, the smoothing effect of the Kalman filter also helps reduce the impact of random noise.

After completing the first-stage integrated navigation calculations, the aircraft’s dynamic motion provides high observability of the triaxial accelerometer’s random zero-bias. Due to the inseparability between the accelerometer zero-bias and the gravity disturbance vector itself, accurately separating the zero-bias is challenging. However, from a frequency perspective, the gravity disturbance vector exhibits relatively complex fluctuations over space (time), while the measurement task for airborne gravity disturbance vectors typically spans several hours. Compared to the accelerometer zero-bias stability, which is measured in months, the random accelerometer zero-bias can be treated as a constant or modeled as a slowly varying function. By performing a weighted least squares estimation based on the values estimated during the integrated navigation filtering process, a relatively accurate estimate of the accelerometer zero-bias can be acquired.

Subtracting the estimated random zero-bias from the accelerometer data and then performing a secondary navigation calculation allows for further separation and decoupling of attitude error-induced coupling errors, accelerometer zero-bias, and the gravity disturbance vector itself. Although the estimated accelerometer bias may include some coupling with the gravity disturbance vector, affecting subsequent calculations of the gravity disturbance vector, this effect is nearly constant and does not impact the overall trend in the gravity disturbance estimates.

### 2.3. Calculation of Gravity Disturbance Vector

The compensated specific force, converted to the *n*-frame from the integrated navigation calculations, along with the position and velocity provided by GNSS and the second derivative of the GNSS position with smoothed motion acceleration, allows for the preliminary gravity disturbance vector $\delta g_{diff}^{n}$ to be obtained via the direct-difference method as described in (1).

The gravity disturbance vector obtained via the direct-difference method contains significant high-frequency noise and residual system errors, which may exhibit a trend due to coupling errors arising from attitude inaccuracies. The global gravity field models, such as EGM2008, which was developed by the National Geospatial-Intelligence Agency (NGA) in collaboration with other institutions, provide a globally consistent and highly precise depiction of the Earth’s gravity field by integrating extensive satellite measurements, ground-based survey data, and other Earth observation data. Although EGM2008 offers gravity anomaly information from the Earth’s surface up to approximately 1000 km in altitude, encompassing gravity potential, terrain correction, and Bouguer anomalies, it is primarily designed to satisfy the needs of low-resolution geodetic applications and thus falls short in capturing local gravity field variations that arise from factors such as changes in rock density, terrain variations, and geological structures.

The external gravitational potential of the Earth exhibits harmonic properties and can therefore be expanded using spherical harmonic functions as follows [12,27]:

(13) $$V(r,\theta ,\lambda )=\frac{GM}{r}\left(1+\sum_{n=2}^{N}\sum_{m=0}^{n}{\left(\frac{R_{e}}{r}\right)}^{n}\left({\bar{C}}_{nm}cos(m\lambda )+{\bar{S}}_{nm}sin(m\lambda )\right)P_{nm}(cos\theta )\right)$$

where G is the gravitational constant, M is the Earth’s mass, and $R_{e}$ is the Earth’s mean radius, approximately 6,378,137 m. r,θ and λ represent the distance from a point outside the Earth to the center of the Earth, the co-latitude, and the longitude, respectively. ${\bar{C}}_{nm}$ and ${\bar{S}}_{nm}$ are the coefficients of the spherical harmonic expansion, and $P_{nm}$ are the associated Legendre polynomials related to the spherical harmonics.

Then, the two horizontal components of the gravity disturbance vector can be obtained by calculating the partial derivatives of the gravitational potential with respect to the distance, namely:

(14) $$\delta g_{E}=\frac{1}{r}\frac{\partial V}{\partial \theta },\delta g_{N}=-\frac{1}{rsin\theta }\frac{\partial V}{\partial \lambda }$$

where $\delta g_{E}$ and $\delta g_{N}$ correspond to the eastward and northward components of the gravity disturbance vector.

The EGM2008 global gravity field model data can be used to provide low frequency about Earth’s gravity field to constrain the trend in the gravity disturbance vector obtained via the direct-difference method. Initially, a large-scale low-pass filter with the frequency of $f_{c1}$ is applied to $\delta g_{diff}^{n}$ to obtain $\delta g_{lb}^{n}$. The low-frequency components in $\delta g_{diff}^{n}$ are then removed to obtain $\delta g_{hb}^{n}$, which is replaced by more reliable model data $\delta g_{model}^{n}$, resulting in the corrected full-band gravity disturbance vector $\delta g_{wb}^{n}$. Finally, a small-scale low-pass filter with cutoff frequency $f_{c2}$ is applied to obtain the final strapdown airborne gravity disturbance vector measurement result.

The filtering scale $N_{l}$ of the large-scale low-pass filter generally depends on the spherical harmonic degree *K* of the chosen gravity field model and the aircraft’s velocity v within the survey area. The specific formula for $N_{l}$ is as follows:

(15) $$N_{l}=\frac{2\pi R_{\varphi }}{\left(K+1\right)v}$$

where $R_{\varphi }$ is the Earth’s radius of curvature at latitude φ, approximately given by $R_{\varphi }=R_{e}\cos\varphi$.

The filtering scale $N_{s}$ of the small-scale low-pass filter depends on the aircraft’s velocity *v* within the survey area and the spatial resolution λ required for the gravity measurement task, also known as the half-wavelength resolution. The specific formula for $N_{s}$ is given by the following:

(16) $$N_{s}=\frac{2\lambda }{v}$$

In general, if λ is chosen to be too large, the strapdown gravity disturbance measurements will more closely approximate the global gravity field’s model curves. However, this also means that high-frequency details of the local gravity field are lost, thereby diminishing the utility of strapdown gravity measurements. Conversely, if λ is chosen to be too small, resulting in a smaller filtering scale, the gravity disturbance measurements will retain more high-frequency noise. This noise can interfere with the high-frequency details of the gravity disturbance vector, making it difficult to distinguish between them and thus affecting accuracy, although it results in higher spatial resolution. Therefore, in practical data processing, a suitable filtering scale is typically determined based on the minimum spatial resolution required for the task and the actual flight speed of the aircraft.

**Figure 2:**
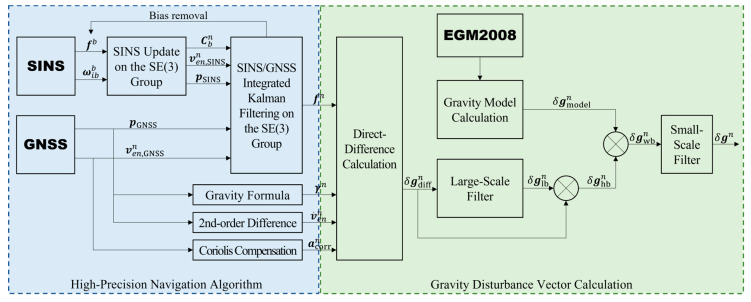
A block diagram of the strapdown airborne gravity disturbance vector measurement method.

The proposed methodological framework for the strapdown airborne gravity disturbance vector measurement is illustrated in Figure 2. This algorithm systematically addresses the principal error sources affecting measurement accuracy.

1. To minimize attitude errors introduced by aircraft maneuvers and air turbulence, thereby reducing the horizontal gravity disturbance coupling errors caused by attitude inaccuracies, an $SE\left(3\right)$ group-based navigation algorithm is employed. This algorithm separates and decouples the motion and errors in the *b*-frame and *n*-frame, and provides a joint representation of their angular and linear motions. y utilizes exponential and logarithmic mappings between the $SE\left(3\right)$ group and its Lie algebra; the algorithm achieves precise compensation for errors due to rotation non-commutativity and coordinate system inconsistency, thereby enhancing attitude accuracy.
2. The accelerometer bias affects both the precise calculation of the aircraft’s linear motion and introduces coupling errors in attitude estimation due to the Coriolis effect. Therefore, this paper first estimates the accelerometer bias through an initial filtering process and models its gradual variation. Subsequently, a second round of navigation computation is performed to mitigate the adverse effects of this bias.
3. The global gravity field information provided by EGM2008 is used to correct the low-frequency trend errors in the direct-difference method results. The preliminary results obtained from the direct-difference method are first corrected by EGM2008 after large-scale filtering, where the filtering scale is determined by the spherical harmonic degree of EGM2008 and the aircraft’s velocity. The corrected results are then further processed with appropriate small-scale filtering to remove high-frequency noise, yielding the final measurement results.

The method proposed addresses several major error sources in strapdown gravity disturbance vector measurements, thereby effectively improving the measurement accuracy.

## 3. Experiment and Results

### 3.1. Data Description

**Figure 3:**
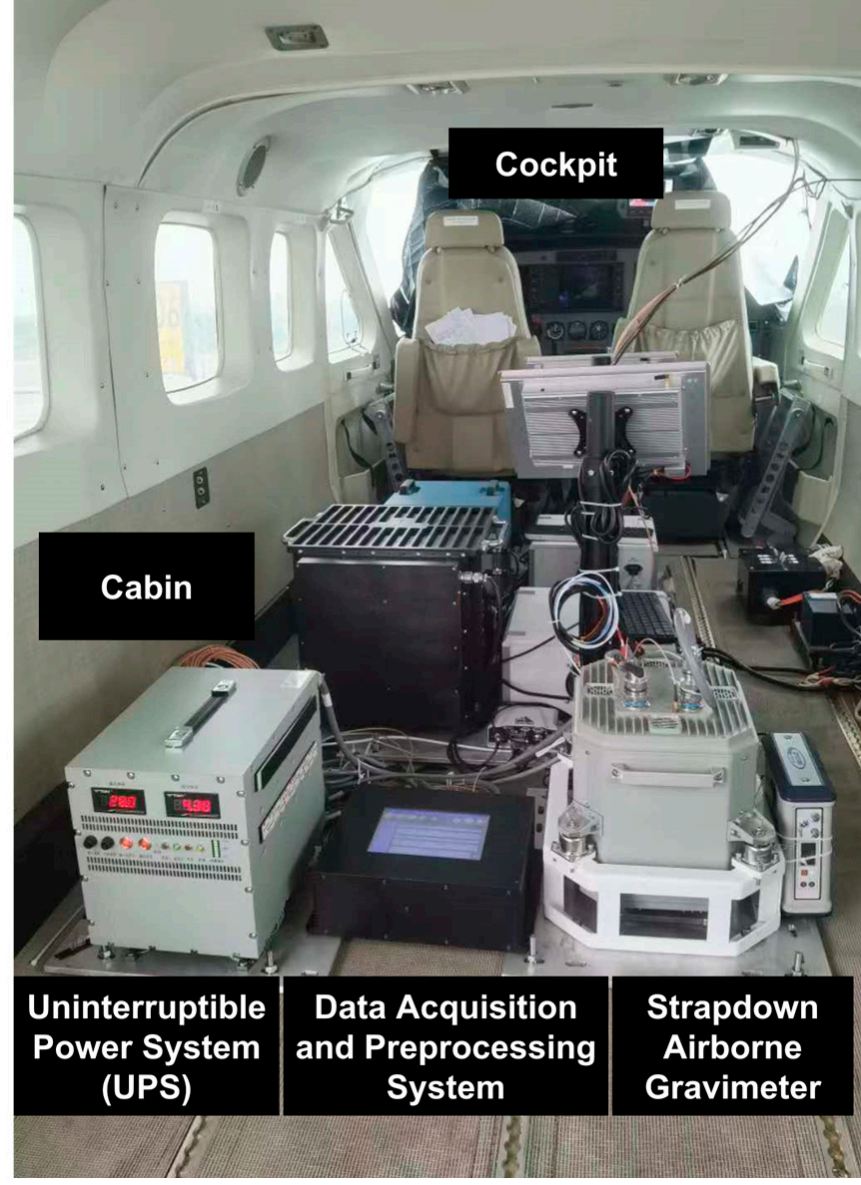
Experimental equipment diagram.

**Figure 4:**
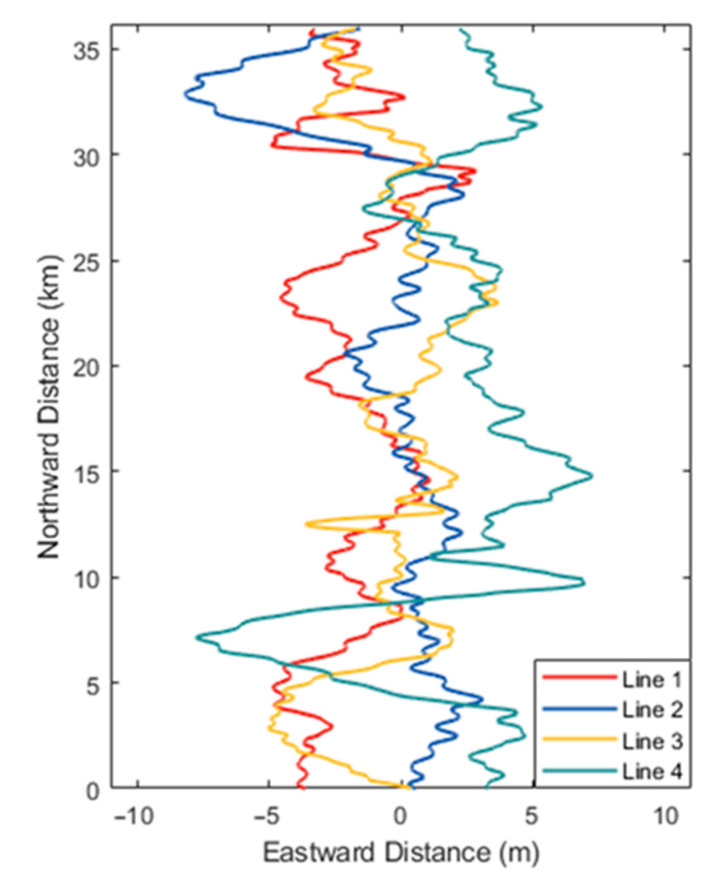
The trajectories of the four north–south repeated survey lines.

To validate the effectiveness of the proposed algorithm for the strapdown airborne gravity disturbance vector, data from a flight experiment conducted in the South China Sea in March 2021 were analyzed. The strapdown vector gravimeter and its data acquisition and preprocessing system, developed and integrated by Southeast University, was mounted on a Cessna 208 fixed-wing aircraft for this measurement task, as shown in Figure 3, and a GNSS receiver was installed on the top of the aircraft to receive satellite signals. Table 1 presents the main performance specifications of the gravimeter, and the gyroscope in the system had a constant bias drift of approximately 0.005°/h, with random noise intensity better than 0.001°/$\sqrt{h}$. Moreover, the GNSS used is based on a differential positioning mode, with a velocity error of approximately 0.005 m/s and a position error of approximately 0.1 m. All inertial sensors’ outputs were synchronized to a frequency of 200 Hz, while the GNSS was recorded at a frequency of 2 Hz. During the experiment, the aircraft initially underwent preliminary calibration while stationary at the airport. It then proceeded through various motion states, including acceleration, ascent, circling, hovering, and constant-speed flight, before entering the gravity measurement area. The aircraft maintained a constant altitude of 650 m and a speed of 60 m/s while performing four north–south repetitive survey lines. The trajectories of the repetitive survey lines are shown in Figure 4.

### 3.2. Data Processing and Results

**Figure 5:**
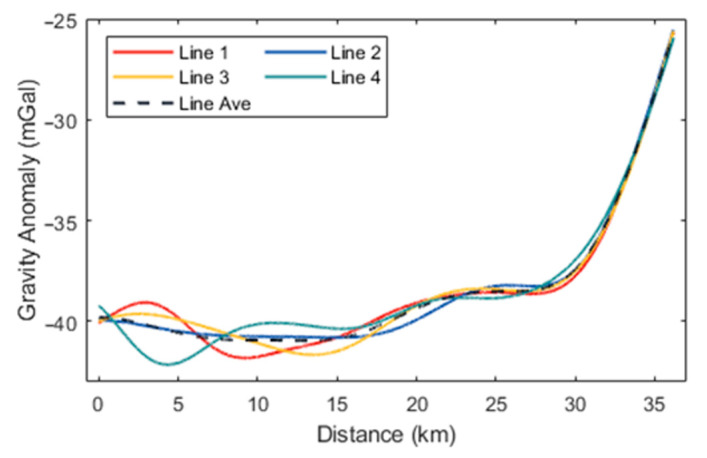
Gravity anomaly curves for the four survey lines.

**Figure 6:**
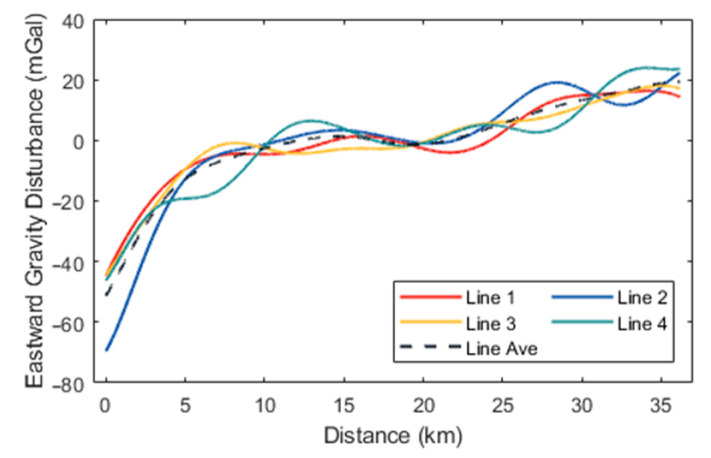
Eastward gravity disturbance curves for the four survey lines.

**Figure 7:**
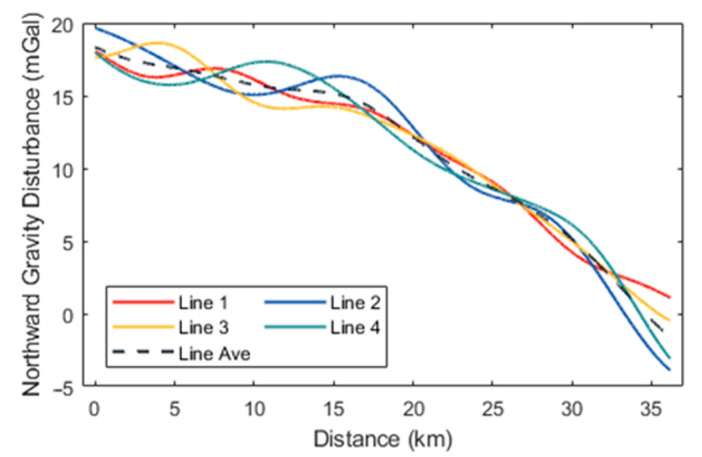
Northward gravity disturbance curves for the four survey lines.

Initially, the high-precision navigation algorithm proposed was employed to preliminarily calculate the aircraft’s attitude and estimate accelerometer bias. At this stage, the horizontal components of the gravity disturbance measurements in different flight directions exhibited significant constant biases, attributable to accelerometer bias. Following the estimation and removal of this bias, the horizontal gravity disturbance components across different flight directions remained consistent. The preliminary gravity disturbance vector measurements obtained using the direct-difference method were subjected to filtering at various scales and compared with data from the 2159th-degree EGM2008 model. According to (15), the spatial resolution of the gravity disturbance reference values provided by EGM2008 within the survey area is approximately 9 km, which corresponds to a low-pass filtering scale of 300 s. Subsequently, a small-scale low-pass filter with a 160-s cutoff was applied to the results incorporating EGM2008 data, yielding the final gravity disturbance vector measurements. The processed flight data are represented by the gravity disturbance vector curves for the four repetitive survey lines, as illustrated in Figure 5, Figure 6 and Figure 7, with internal consistency accuracy detailed in Table 2. The results demonstrate that the gravity anomaly achieved with the proposed method is superior to 0.5 mGal, with an internal consistency accuracy of approximately 0.85 mGal for the northward gravity disturbance component and about 4.0 mGal for the eastward gravity disturbance component, corresponding to a spatial resolution of 4.8 km. Additionally, Table 3 presents the internal consistency accuracy of gravity disturbance vector measurements corresponding to different filter scale of 60 s, 100 s, and 250 s, respectively.

### 3.3. Comparison

The primary methods for solving the gravity disturbance vector include the aforementioned direct-difference method [6,7,9,23,28,29], the optimal estimation method based on Gaussian–Markov stochastic process modeling [11,17,30], and the optimal estimation method based on two-dimensional planar field modeling [16,31].

In the direct-difference method, the aircraft’s attitude is first determined using a traditional 15-state SINS/GNSS integrated navigation filter to obtain the specific force in the *n*-frame. Then, the preliminary gravity disturbance vector measurements, containing substantial high-frequency noise and significant low-frequency trends, are obtained via direct-difference, as shown in (1). Subsequently, a low-pass filter is applied to remove high-frequency noise, followed by the endpoint-matching method or WCF to correct low-frequency trend errors.

In the optimal estimation method based on Gaussian–Markov modeling, the gravity disturbance vector is typically modeled as a second-order (or third-order) Gaussian–Markov stochastic process driven by zero-mean Gaussian white noise, as shown in Equations (17) and (18) [30,32]. The gravity disturbance vector and its intermediate modeling variables are augmented into the state vector, and estimation is performed using Kalman filtering and smoothing methods, as follows:

(17) $$\mathrm{Second}‐\mathrm{order}:\;\delta {\ddot{g}}^{n}+2\beta \delta {\dot{g}}^{n}+\beta^{2}\delta g^{n}=q$$

(18) $$\mathrm{Third}‐\mathrm{order}:\;\delta {\dddot{g}}^{n}+3\beta \delta {\ddot{g}}^{n}+3\beta^{2}\delta {\dot{g}}^{n}+\beta^{3}\delta g^{n}=q$$

where β is a correlation parameter determined by the prior correlation length of the gravity disturbance and q is the white noise vector with variance $\sigma_{q}^{2}=4\beta^{3}\sigma_{\delta g}^{2}$ for the second-order modeling and $\sigma_{q}^{2}=\frac{16}{3}\beta^{5}\sigma_{\delta g}^{2}$ for the third-order modeling, where $\sigma_{\delta g}^{2}$ represents the prior variance of the gravity disturbance.

In the optimal estimation method based on two-dimensional planar field modeling, gravity measurements are usually conducted over a local plane in a grid pattern. The gravity disturbance signal is modeled as a two-dimensional Gaussian–Markov stochastic process or expanded using spherical harmonic functions and radial basis functions. The model parameters are then augmented into the state vector, with estimation performed via Kalman filtering and smoothing methods.

**Figure 8:**
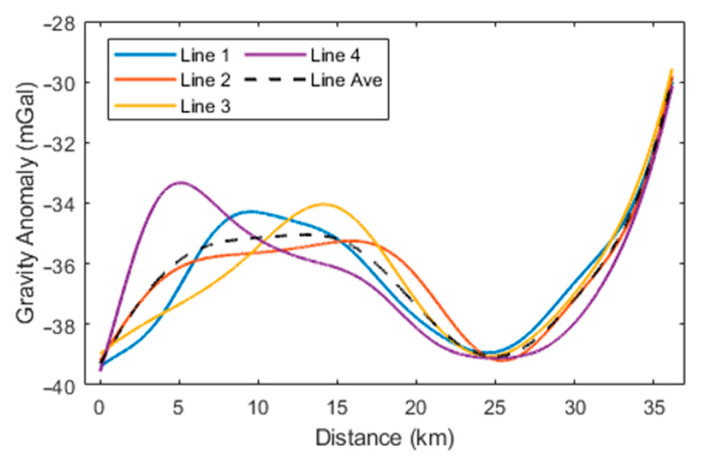
Gravity anomaly curves for the four survey lines (the comparative method).

**Figure 9:**
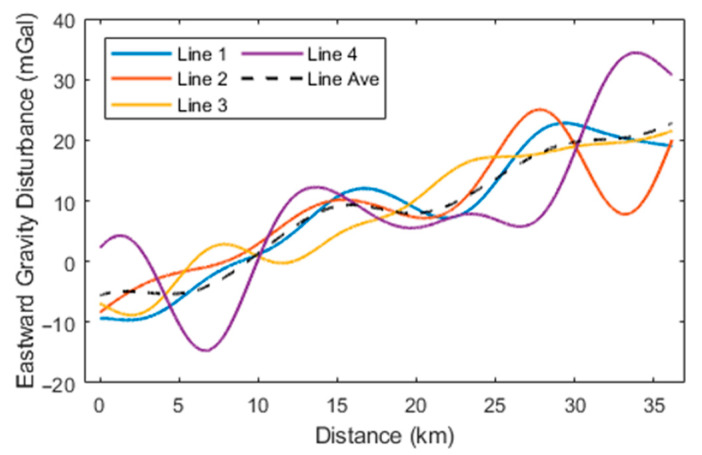
Eastward gravity disturbance curves for the four survey lines (the comparative method).

**Figure 10:**
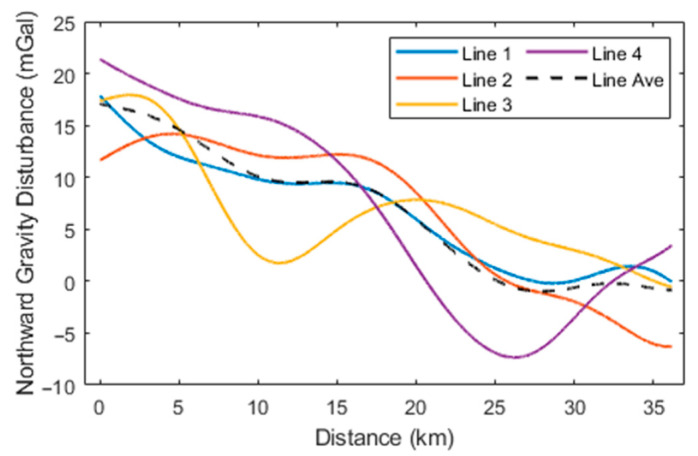
Northward gravity disturbance curves for the four survey lines (the comparative method).

In this experiment, a single-track repeated measurement method was adopted, making the planar field modeling approach inapplicable. Gaussian–Markov modeling is based on the assumption of uniform and isotropic local gravity variations, while the actual gravity disturbance may deviate somewhat from this assumption. Additionally, Gaussian–Markov modeling of the gravity disturbance vector is vulnerable to prior gravity parameters such as correlation length and variance, potentially leading to differences in the results. By comparison, the direct-difference method remains a widely used and effective approach. Therefore, the subsequent analysis will focus on comparing the proposed method with the traditional direct-difference method. The specific data processing procedure is as follows: After processing the experimental data through the traditional 15-state SINS/GNSS integrated navigation system and the direct-difference method, the final results were obtained using a 160-s FIR low-pass filter and endpoint matching. Figure 8, Figure 9 and Figure 10 show the gravity disturbance vector curves calculated using this method, and the internal consistency accuracy of this comparative method, along with the proposed method in this paper, is listed in Table 4.

The results from this comparative method are inferior to those of the proposed method. This is mainly because the high-precision navigation algorithm used in this paper, despite the increased processing time, provides more accurate aircraft attitude estimates, mitigating the coupling effect between attitude errors and the vertical gravity acceleration on the gravity disturbance vector measurement, especially for the two horizontal components. Additionally, the fitting and correction of accelerometer biases, along with the low-frequency trend correction using EGM2008, more accurately and reasonably remove the low-frequency systematic errors present in the measurements compared to the endpoint-matching method.

## 4. Conclusions

This paper introduces a novel method for strapdown airborne gravity disturbance vector measurement. This approach employs the geocentric inertial coordinate system as the reference and updates the motions of both the body and local geographic coordinate systems using Lie groups, while also propagating their motion errors. This procedure yields accurate attitude estimates and preliminary accelerometer bias estimates. Subsequently, navigation calculations are refined to obtain precise specific force measurements in the local geographic coordinate system, accounting for the estimated accelerometer bias. The direct-difference results are filtered and compared with the EGM2008 gravity model to establish an appropriate cutoff frequency for the model data, which is used to correct low-frequency trends. A final small-scale low-pass filter is applied to achieve the ultimate gravity disturbance vector measurements. Results from processing flight experiment data indicate that the method achieves internal consistency accuracies better than 0.5 mGal for the gravity anomaly, approximately 0.85 mGal for the northward gravity disturbance component, and about 4.0 mGal for the eastward gravity disturbance component, with a spatial resolution of 4.8 km, and the corresponding low-pass filter scale is 160 s.

## Figures and Tables

**Table 1:** Main performance specifications of the strapdown vector gravimeter.

Index Name	Index Parameter
Dynamic range	±2.0 g
Static measurement accuracy	<0.4 mGal
Dynamic measurement accuracy	<1.5 mGal

**Table 2:** Internal consistency accuracy of the gravity disturbance vectors for the four survey lines.

Lines	Gravity Anomaly ^1^	Eastward Gravity Disturbance ^1^	Northward Gravity Disturbance ^1^
Line 1-2-3-4	0.44	4.04	0.85
Line 1-2-3	0.28	3.55	0.78
Line 1-2-4	0.45	4.24	0.83
Line 1-3-4	0.49	3.14	0.74
Line 2-3-4	0.41	4.20	0.84
Line 1-2	0.29	3.57	0.81
Line 1-3	0.22	1.69	0.47
Line 1-4	0.52	3.14	0.63
Line 2-3	0.21	3.55	0.69
Line 2-4	0.34	4.23	0.71
Line 3-4	0.48	3.07	0.78

^1^ (Units: mGal, $1mGal=10^{-5}m/s^{2}$).

**Table 3:** Internal consistency accuracy and spatial resolution of the gravity disturbance vector measurements for the four survey lines corresponding to different filter scales.

Filter Scale	Gravity Anomaly ^1^	Eastward Gravity Disturbance ^1^	Northward Gravity Disturbance ^1^	Spatial Resolution
60-s	2.70	20.06	2.13	1.8 km
100-s	1.09	9.08	1.34	3.0 km
250-s	0.20	3.73	0.53	7.5 km

^1^ (Units: mGal).

**Table 4:** Internal consistency accuracy and spatial resolution of the gravity disturbance vector measurements for the four survey lines corresponding to different methods.

Methods	Gravity Anomaly ^1^	Eastward Gravity Disturbance ^1^	Northward Gravity Disturbance ^1^	Spatial Resolution
Proposed method	0.44	4.04	0.85	4.8 km
The comparative method	0.71	4.66	3.33	4.8 km

^1^ (Units: mGal).

## Data Availability

The dataset is not publicly available because it involves sensitive information, but is available from the corresponding author on reasonable request.
